# IB, NF-B Regulation Model: Simulation Analysis of Small Number of Molecules

**DOI:** 10.1155/2007/25250

**Published:** 2008-01-24

**Authors:** Anamika Sarkar, Marina Meila, Robert B Franza

**Affiliations:** 1 Bioengineering Department, Cell Systems Initiative, University of Washington, Seattle, WA 98195, USA; 2 Department of Pharmacology and Systems Therapeutics, Mount Sinai School of Medicine, P.O. Box 1215, One Gustave L. Levy Place, New York, NY 10029, USA; 3 Statistics Department, University of Washington, Seattle, WA 98195, USA

## Abstract

The regulation of IB, NF-B is of foremost interest in biology as the transcription factor NF-B has multiple target genes. We have modeled a previously published model by Hoffmann et al. (2002) of IB, NF-B mathematically as discrete reaction systems. We have used stochastic algorithm to compare the results when there are large and small numbers of molecules available in a finite volume for each protein. Our results for small number of molecules show that with continuous presence of stimulation, nuclear NF-B oscillates continuously in every individual cell rather than damping, which was observed in cell population results. This characteristic of the system is missed when averaged behavior is studied.

## 

[1,2,3,4,5,6,7,8,9,10,11,12,13,14,15,16,17,18,19,20,21,22,23,24,25,26,27,28,29,30,31,32,33,34,35,36]
